# Methyl-Beta-Cyclodextrin Restores Aberrant Bone Morphogenetic Protein 2-Signaling in Bone Marrow Stromal Cells Obtained from Aged C57BL/6 Mice

**DOI:** 10.3390/jdb12040030

**Published:** 2024-11-18

**Authors:** Daniel Halloran, Venu Pandit, Kelechi Chukwuocha, Anja Nohe

**Affiliations:** Department of Biological Sciences, University of Delaware, Newark, DE 19716, USA; hallorand@chop.edu (D.H.); vpandit@udel.edu (V.P.); kelechib@udel.edu (K.C.)

**Keywords:** osteoporosis, osteoblasts, BMP-2, BMPRIa, C57BL/6 mice, MβCD, QDot^®^s

## Abstract

During aging, disruptions in various signaling pathways become more common. Some older patients will exhibit irregular bone morphogenetic protein (BMP) signaling, which can lead to osteoporosis (OP)—a debilitating bone disease resulting from an imbalance between osteoblasts and osteoclasts. In 2002, the Food and Drug Administration (FDA) approved recombinant human BMP-2 (rhBMP-2) for use in spinal fusion surgeries as it is required for bone formation. However, complications with rhBMP-2 arose and primary osteoblasts from OP patients often fail to respond to BMP-2. Although patient samples are available for study, previous medical histories can impact results. Consequently, the C57BL/6 mouse line serves as a valuable model for studying OP and aging. We find that BMP receptor type Ia (BMPRIa) is upregulated in the bone marrow stromal cells (BMSCs) of 15-month-old mice, consistent with prior data. Furthermore, conjugating BMP-2 with Quantum Dots (QDot^®^s) allows effective binding to BMPRIa, creating a fluorescent tag for BMP-2. Furthermore, after treating BMSCs with methyl-β-cyclodextrin (MβCD), a disruptor of cellular endocytosis, BMP signaling is restored in 15-month-old mice, as shown by von Kossa assays. MβCD has the potential to restore BMPRIa function, and the BMP signaling pathway offers a promising avenue for future OP therapies.

## 1. Introduction

During the natural aging process of humans, the body undergoes senescence and alterations in signaling pathways. As such, the development of several diseases, including metabolic disorders, neurocognitive disorders, and bone disorders such as osteoporosis (OP) can occur [1,2,3]. While the process of aging has been investigated, the precise molecular and cellular aberrancies that are presented during this process are unclear. As the bone morphogenetic protein (BMP) signaling pathway is altered in patients diagnosed with OP, it can be explored to determine the role of BMP-2 in aging and if these effects can be reversed in the bones [4,5,6].

OP is a devastating bone disorder that affects more than 10 million Americans, and 80% of those diagnosed are women [7,8,9]. OP is a drastic decrease in bone mineral density (BMD) that is likely caused by an imbalance between osteoblasts (bone-forming cells) and osteoclasts (bone-resorbing cells) [10,11]. Furthermore, OP is extremely costly, decreases the quality of life for patients, is associated with increased mortality, and is currently incurable [7,12,13].

The current treatment options are commonly anabolic, which restores bone loss, or anti-resorptive, which reduces bone degradation [14,15,16,17,18,19,20,21]. However, each therapeutic has been associated with adverse side-effects which include osteolysis, hematoma formation, necrosis, and others [8,22,23,24,25,26,27]. In addition, there is currently only one drug approved by the Food and Drug Administration (FDA) that targets osteoblasts and osteoclasts simultaneously, and this is known as Romosozumab [28,29,30,31,32,33]. This therapeutic is a sclerostin antibody and induces osteogenesis while limiting bone degradation. Its entire mechanism is currently being deciphered, especially as its long-term effects are unclear. Furthermore, the side-effects of Romosozumab have been reported and include hepatitis and osteonecrosis [28,29,30,31,32,33]. Moreover, a treatment option that safely treats OP without adverse side-effects and targets osteoblast and osteoclast activity is desperately needed.

A pathway that may be targeted for the treatment of OP is BMP signaling. BMPs are members of the transforming growth factor-β (TGF-β) superfamily and regulate many cellular and molecular processes [34,35,36,37]. Specifically, BMP-2 was identified in 1965 as a crucial growth factor that regulates osteogenesis during development and in adulthood [38,39]. BMP-2 also regulates many other pathways such as chondrogenesis, adipogenesis, cardiogenesis, somite formation, and limb development [40,41,42,43,44,45,46,47,48]. To activate these pathways, BMP-2 binds with high affinity to BMP receptor type Ia (BMPRIa) localized in caveolae enriched with caveolin-1 alpha (Cav1 α) isoforms or within clathrin-coated pits (CCPs) [49,50,51,52,53,54]. Afterward, BMP receptor type II (BMPRII) is recruited to the membrane or is already localized to the membrane domains with BMPRIa as pre-formed complexes containing the Cav1 αβ isoform [49,50,53,54]. BMPRII phosphorylates BMPRIa at its glycine–serine (GS) box and causes the release of protein kinase CK2 (CK2) [55]. The release of CK2 from BMPRIa allows the receptor to phosphorylate downstream proteins including SMAD1, 5, and/or 8 in the canonical signaling pathway [5,47,51,52,54,56]. The p-SMADs recruit SMAD4 and the entire complex translocates to the nucleus to serve as transcription factors for osteogenesis. In the non-canonical pathway, BMPRIa activates the mitogen-activated protein kinase (MAPK) and phosphatidylinositol 3-kinase (PI3K) signaling pathways to increase cellular survival and proliferation [57,58,59,60].

As BMP-2 is essential for osteogenesis, the FDA approved it as a bone regeneration therapeutic during anterior lumbar interbody fusion (ALIF) surgery and facial reconstruction in 2002 [61]. Shortly after its appearance in the clinic, several side-effects were reported, and the primary osteoblasts isolated from patients diagnosed with OP do not respond positively to this treatment, even though these cells produce more BMPRIa protein than control cells [6,62,63,64,65]. As such, the usefulness of BMP-2 is contradictory and must be used with extreme caution. However, the BMP signaling may still be utilized in future medical interventions without exogenous BMP-2.

As receptor localization determines BMP signaling, it is plausible that BMPRIa and/or BMPRII may be mis-localized in patients diagnosed with OP. Recent data demonstrate that after primary osteoblasts isolated from OP patients are stimulated with BMP-2 treatment, there is no increase in canonical or non-canonical signaling, and mineralization is not induced [5,6]. This lack of signaling may be caused by the improper localization or shuttling of BMPRs, which prevents BMP-2 activity. However, the precise role and binding of BMP-2 to BMPRs in these cells is currently unknown. A current pharmacological agent that can disrupt receptor localization and prevent endocytosis for short time-frames is methyl-beta-cyclodextrin (MβCD) [66,67]. It is possible that after treating cells with MβCD and then BMP-2, BMPRIa and/or BMPRII can be re-localized to their proper location to restore signaling. As MβCD produces transient responses, the potential re-shuttling of receptors is temporary and may cause the permanent relocation of BMPRIa to restore BMP signaling. To uncover the possible improper binding of BMP-2 to its receptors in these cells, this protein can be conjugated to Quantum Dot^®^s (QDot^®^s) to determine its mode of action. QDot^®^s are photostable molecules that photo-bleach less than other traditionally used dyes [68,69,70,71,72]. Furthermore, QDot^®^s can be carboxylated to allow for bond formation with peptides or proteins [73,74]. In addition, while primary osteoblasts isolated from OP patients are an effective method to study the disease, several complications arise including previous medical history, genetic differences, and comorbidities. Thus, a model that eliminates these factors must be explored. One model that can be utilized to overcome these obstacles is the C57BL/6 (B6) mouse. These mice display low peak BMD, are readily susceptible to bone disorders, and recent data demonstrate that they are a comparable model to study human OP [75,76,77]. Furthermore, the localization of BMP-2 in the bone marrow stromal cells (BMSCs) within these mice is unknown and requires further attention to determine if these cells can serve as a reservoir to produce osteoblasts.

Here, we first investigated the expression of BMPRIa at both 6 months and 15 months in the BMSCs of female B6 mice and evaluated if these data are representative of previous data obtained from OP patients. Next, we explored the co-localization of a BMP-2-QDot^®^s probe with BMPRIa in the BMSCs of the 6- and 15-month-old mice with or without MβCD. Finally, we observed the production of a mineralized matrix by these cells after BMP-2 stimulation with or without MβCD. Altogether, these data demonstrate that MβCD is able to improve BMP signaling, suggesting that the receptors are mis-localized on the plasma membrane in aged mice that are reflective of OP patients. These results can inform future therapeutics that are desperately needed to treat OP.

## 2. Materials and Methods

### 2.1. Mice Acquisition and Ethical Approval

C57BL/6 (B6) female mice aged 6 (*N* = 20) and 15 months (*N* = 20) were obtained from Charles River Laboratories (Horsham, PA, USA). The mice were housed at the University of Delaware in the Life Sciences Research Facility (University of Delaware, Newark, DE, USA) and acclimated to the new environment for one week. Each mouse had access to food and water, and they were housed as 4–5 mice of the same age per cage. All the research conducted in this study was approved by the Institutional Animal Care and Use Committee (University of Delaware, Newark, DE, USA): AUP #1194.

### 2.2. Organ and Cell Isolation

The B6 mice were euthanized with CO_2_ and their femurs were extracted. Each femur was sliced at the distal and proximal femoral heads and then flushed three times with alpha minimum essential media (αMEM; Caisson Labs, Smithfield, UT, USA) to isolate bone marrow stromal cells (BMSCs). Afterward, the flushed solutions were filtered with 70 μM cell strainers (Stellar Scientific, Baltimore, MD, USA) into 50 mL conical tubes (Cole-Palmer, Vernon Hills, IL, USA) and centrifuged at 1500 rotations per minute (RPM) for 5 min at 4 °C. The supernatant was aspirated and the cell pellets were resuspended with αMEM supplemented with 10% fetal bovine serum (FBS; Gemini Bioproducts, West Sacramento, CA, USA), 1% penicillin/streptomycin (pen/strep; Fisher Scientific, Pittsburg, PA, USA), and 1% antibiotic/antimycotic (anti/anti; Gemini Bioproducts, West Sacramento, CA, USA). The cell densities were counted and the cells were plated as noted by each method.

### 2.3. Immunofluorescent Staining

#### 2.3.1. Staining of BMPRIa of Unstimulated (US) B6 Mice

The BMSCs isolated from the femurs at 6 and 15 months and 1 × 10^6^ total cells were seeded in 12-well plates (Nest Scientific, Woodbridge Township, NJ, USA) on 18 mm diameter rounded coverslips (Catalog #CS-R18-100, Amscope, Irvine, CA, USA). The cells were grown in αMEM supplemented with 10% FBS, 1% pen/strep, and 1% anti/anti for 7–10 days. Every third day, fresh media was replenished. On the final day of cell culture, the media was aspirated and the cells were washed with ice-cold 1X phosphate-buffered saline (PBS) and fixed with 4.4% paraformaldehyde (PFA, pH 7.2; Sigma Aldrich, St. Louis, MO, USA) for 15 min at room temperature. Afterward, the cells were washed three times with ice-cold 1X PBS. The cells were permeabilized with 0.1% Saponin (Sigma-Aldrich, St. Louis, MO, USA) dissolved in 1X PBS for 10 min and then blocked for 1 h on ice with 3% bovine serum albumin (BSA, Fisher Scientific, Pittsburgh, PA, USA) and 0.1% Saponin diluted in 1X PBS. The cells, except for those in the secondary control condition, were incubated with 1:100 dilutions of a primary rabbit polyclonal BMPRIa antibody (Catalog #100743-T08; Sino Biological, Beijing, China) in 1X PBS, 3% BSA, and 0.1% Saponin for 1 h on ice. The cells were washed three times with ice-cold 1X PBS and all the experimental groups were incubated with 1:500 solutions of a secondary chicken-anti-rabbit antibody (Alexa Fluor^TM^594, Catalog #A21442; Invitrogen, Eugene, OR, USA) in 1X PBS, 3% BSA, and 0.1% Saponin for 1 h on ice away from light. The cells were washed three times with ice-cold 1X PBS and the nuclei were stained with Hoechst 33,342 (Catalog #AR0039, Bolster Bio, Pleasanton, CA, USA) for 7.5 min. The cells were washed once with 1X PBS and the coverslips were mounted onto glass slides with Airvol. After drying, the slides were imaged using the Zeiss LSM880 confocal microscope with Airyscan (Wolf Hall, University of Delaware, Newark, DE, USA) with the 63x objective lens. Z-stack images (64 slices) were acquired at 0.3 μm per section with a pinhole of 0.25 Airy Units (AU). The images were processed using the Huygens deconvolution software with the point spread function (PSF) applied for enhanced clarity and resolution. Each experiment was performed in triplicate, with 10 images acquired per condition, and data normalized to the secondary control. All the data were analyzed and processed in ImageJ (NIH, Bethesda, MD, USA).

#### 2.3.2. Staining of Cells Treated with Methyl-β-Cyclodextrin and BMP-2-QDot^®^s

Similarly to the above, the BMSCs were isolated from the femurs of the B6 mice [78]. The cells were plated and on day 6, they were treated with 100 µM of methyl-β-cyclodextrin (MβCD; Catalog #M1356, TCI America, Montgomeryville, PA, USA) or left US for 24 h. The cells were then treated with 40 nM of a BMP-2-QDot^®^s conjugation for 6 h or left US as previously described [73]. The cells were subjected to immunostaining as described in Section 2.3.1. The cells were imaged using confocal microscopy using the 63X objective lens and an additional magnification of 10 to focus on BMPRIa.

### 2.4. Lysate Collection

As described before, the BMSCs were obtained from the femurs of the 6- and 15-month-old B6 mice. In total, 1.5 × 10^7^ total cells were seeded into 6-well plates and grown in αMEM. The cells were washed three times with ice-cold 1X PBS and scraped into 300 µL of Radioimmunoprecipitation Assay (RIPA) lysis buffer that contained 0.44 g NaCl, 1 mL TritonX-100 (Acros Organics, Geel, Belgium), 0.5 g Sodium Deoxycholate (Thermo Scientific, Rockford, IL, USA), 12.5 mL 0.5 M Tris Buffer pH 6.8, 0.05 g Sodium Dodecyl Sulfate (SDS, Fisher Scientific, Geel, Belgium), and 36.5 mL sterile H_2_O. Furthermore, 1 mM (PMSF, MP Biomedicals, Solon, Ohio, USA) and 1 mM protease inhibitor (PI) cocktail (Thermo Scientific, Rockford, IL, USA) were added to the RIPA buffer. After incubation in RIPA for 10 min on ice, the lysates were transferred to 1.5 mL microcentrifuge tubes and sonicated (Cleanosonic, Branson Digital Sonifier, Richmond, VA, USA) at 21% amperes for 20 s, three times. The lysates were centrifuged at 12,700 RPM for 10 min at 4 °C and the supernatants were collected. Protein estimation was conducted with the Pierce^TM^ BCA Protein Assay Kit (Thermo Scientific, Rockford, IL, USA) and analyzed with the NanoDropTM One/OneC Microvolume Spectrophotometer (Thermo Fisher Scientific, Waltham, MA, USA). All the lysates were normalized to the lowest protein concentration before loading into the gels.

### 2.5. Western Blotting

The lysates were boiled for 10 min at 95 °C and 20 µL of each were loaded into a 10% SDS-polyacrylamide (SDS-PAGE) gel. Gel electrophoresis was carried out at 120 V for 1 h and 30 min and the proteins were transferred to a PVDF (Immobilon, Darmstadt, Germany) membrane for 1 h and 10 min at 15 V using the semi-dry transfer machine (BioRad, Hercules, CA, USA). The membrane was blocked with 5% BSA diluted in 1X Tris-Buffered Saline-Tween (TBST) for 1 h on ice. The blot was incubated with a 1:1000 dilution of BMPRIa (same as above) diluted in 1X TBST supplemented with 1% BSA overnight at 4 °C. On the following day, the blot was washed three times with 1X TBST and incubated with a 1:5000 dilution of goat-anti-rabbit IgG-HRP (ab6721, Abcam, Cambridge, UK) secondary antibody diluted in 1X TBST with 1% BSA for 1 h. The blots were washed three more times with 1X TBST and treated with SuperSignalTM West Pico Plus Chemiluminescent Substrate (Thermo Scientific, Rockford, IL, USA) for five minutes. All the protein bands were detected with the Invitrogen iBright1500 machine (Thermo Fisher Scientific, Waltham, MA, USA)

### 2.6. Von Kossa Assay

The BMSCs were isolated from the femurs of the 6- and 15-month-old B6 female mice. A total of 500,000 total cells were plated in 24-well plates in αMEM for six days. The cells were supplemented with 1% pen/strep, 1% anti/anti solution, 200 μL of 25 mg/mL ascorbic acid (Fisher Scientific, Fair Lawn, NJ, USA), and 800 μL of 0.22 g/mL β-glycerol phosphate (Alfa Aesar, Ward Hill, MA, USA). On day 6, the cells were treated with 100 µM MβCD for 24 h or left US. On the following day, the cells were treated with 40 nM BMP-2 or left US for 5 days. The media was changed and stimulations were replaced on day 3. On day 5, the media was aspirated, the plates were washed with 1X PBS, and the cells were fixed with 4.4% PFA at room temperature for 15 min. The wells were washed three times with 1X PBS and incubated with 5% silver nitrate (Science Company, Lakewood, CO, USA) dissolved in DiH_2_O for 1 h under UV light. The silver nitrate was removed and the cells were washed with DiH_2_O until excess solute was removed. After drying for two days, random images were acquired and data were analyzed in ImageJ. All the data were normalized to the US groups for both ages.

### 2.7. Statistical Analysis

Chauvenet’s criterion test was employed to remove any outliers from all the experimentation. The data were statistically analyzed using the single factor analysis of variance (ANOVA) and the Tukey–Kramer HSD statistical tests. The error bars above the bars in the graph are representative of the standard error of the mean (SEM). Statistical significance was set to *p* ≤ 0.05 and is displayed as lettering; here, the letters “a, b, c”, etc., represent group one as the letter “a”, group two corresponds to the letter “b”, and so on. For example, if there is a letter “a” above a bar, that means this group is statistically different from group one.

## 3. Results

### 3.1. The 15-Month-Old B6 Mice Produce More BMPRIa Compared to 6-Month-Old Mice via Western Blotting

**Figure 1:**
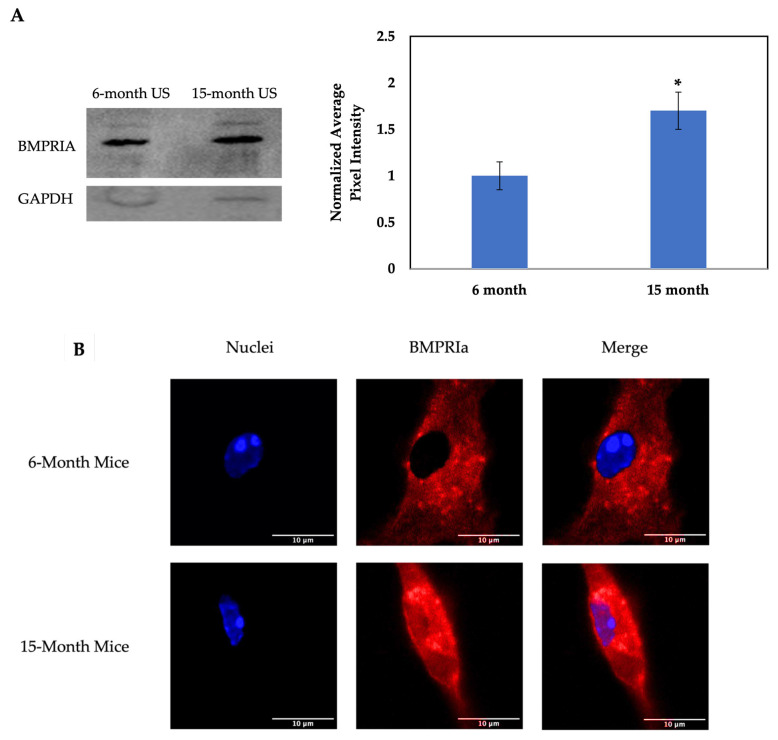
Increased protein detection of BMPRIa in the BMSCs isolated from the 15-month-old mice compared to the control 6-month-old B6 mice. The BMSCs were isolated from the femurs of the 6- and 15-month-old B6 mice. The cells were grown, without additional stimulation, for 10–12 days. (**A**) Lysates were collected and probed for BMPRIa. Protein concentration was normalized and GAPDH was used as the loading control. The protein concentration was quantified via densitometry measurements and statistical significance is displayed by the “*”. The *p*-value was set to 0.05 and the significance was calculated with Student’s *t*-test. (**B**) BMPRIa was detected via immunofluorescent staining and images were acquired with confocal microscopy. Representative images are displayed and the scalebars are set to 10 µm.

**Figure 2:**
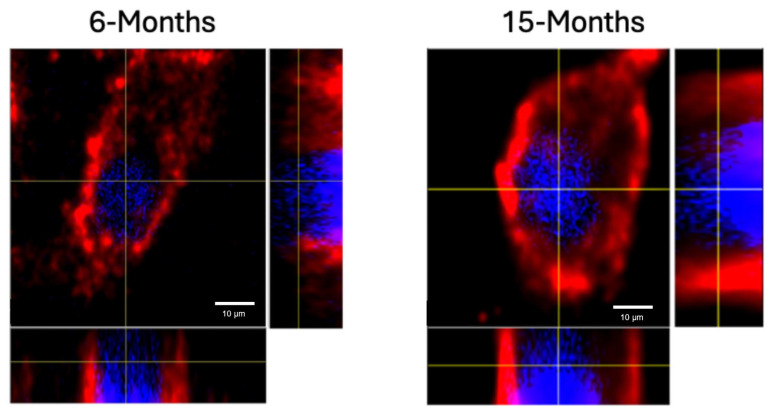
Representative high-resolution z-stack images showing BMPRIa localization in the orthogonal view from the US BMSCs isolated from the 6- and 15-month-old C57BL/6 mice. Immunofluorescence staining was performed using an anti-BMPRIa antibody. BMPRIa is shown in red, and nuclei are stained with Hoechst dye (blue). The images were captured at 63× magnification; scale bar = 10 μm. The orthogonal view provides slices through different regions of the cell, allowing for the precise visualization of BMPRIa localization. BMPRIa is observed to be localized to the plasma membrane in BMSCs from both age groups (6 and 15 months).

It was recently demonstrated that primary osteoblasts isolated from OP patients produce more BMPRIa protein compared to control patients via immunofluorescent staining and Western blotting [6]. Additionally, the increased detection of BMPRIa is also observed in the BMSCs isolated from the 15-month-old mice compared to the 6-month-old mice as indicated by confocal microscopy [75]. Furthermore, we isolated lysates from the US BMSCs of the 6- and 15-month-old mice and detected them for BMPRIa. As noted in Figure 1, the 15-month-old mice produce more BMPRIa compared to the 6-month-old mice, confirming the results obtained in previous studies. Next, we determined if BMP-2-QDot^®^s were able to bind to BMPRI, or if treatment with MβCD was necessary to re-establish proper signaling. Utilizing high-resolution z-stack images in orthogonal viewpoints, BMPRIa was indeed upregulated in the 15-month-old mice and confined to the plasma membrane (Figure 2).

### 3.2. MβCD Improves the Binding of BMP-2-QDot^®^s to BMPRIa in BMSCs Isolated from 15-Month-Old B6 Mice

**Figure 3:**
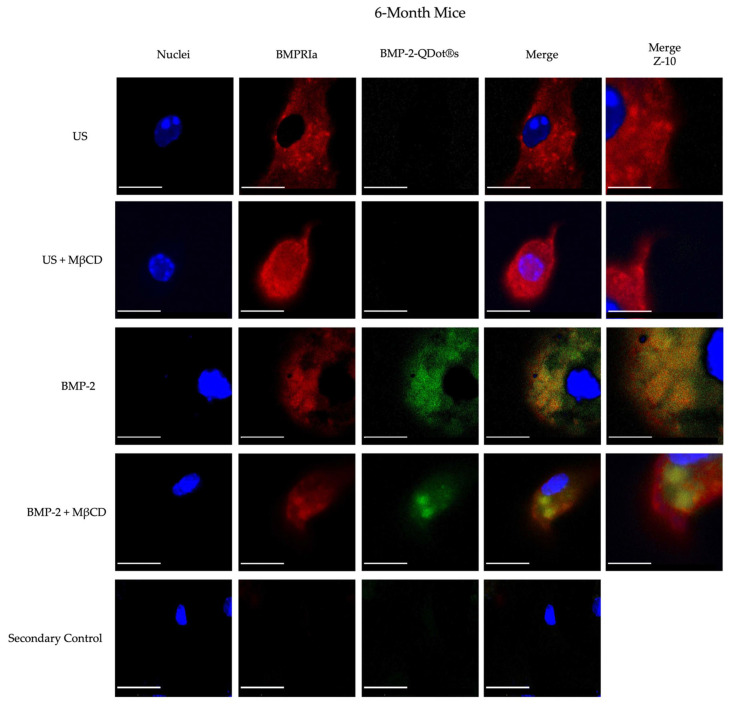
Immunostaining of the BMSCs isolated from the 6-month-old female B6 mice. The cells were obtained from the 6-month-old mice and stimulated with BMP-2-QDot^®^s, MβCD, or left US. The cells were immunostained and observed with confocal microscopy. Representative images are displayed, and the scale bars are set to 10 μm. Z-10 images are obtained with a 63x objective and magnified 10× to observe the precise localization of BMPRIa or BMP-2 and the scale bars are set to 1 μm.

**Figure 4:**
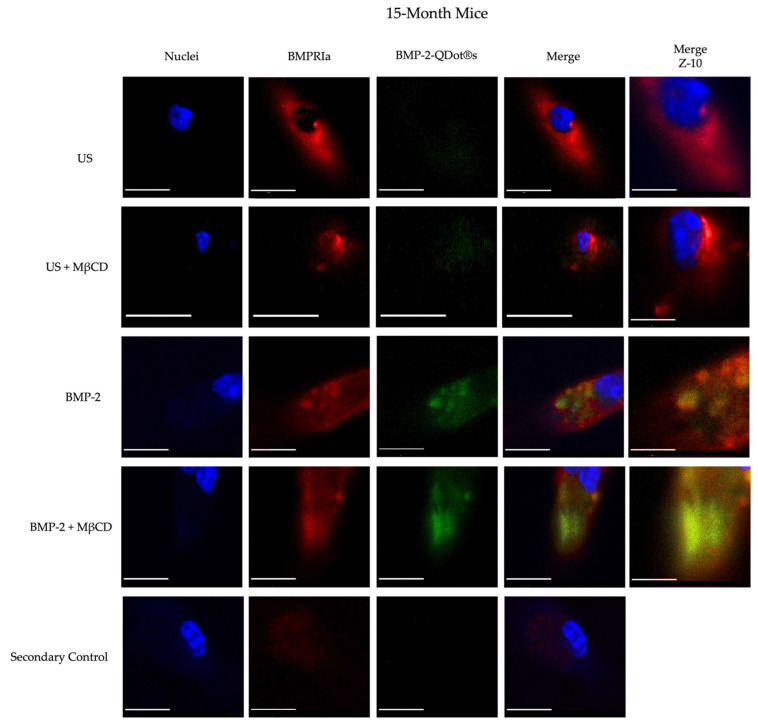
Immunostaining of the BMSCs isolated from the 15-month-old female B6 mice. The cells were obtained from the 15-month-old mice and stimulated with BMP-2-QDot^®^s, MβCD, or left US. The cells were immunostained and observed with confocal microscopy. Representative images are displayed, and the scale bars are set to 10 μm. Z-10 images are obtained with a 63x objective and magnified 10× to observe the precise localization of BMPRIa or BMP-2 with the scale bars set to 1 μm.

To further understand the biological function of BMP-2 in the BMSCs of 15-month-old female B6 mice, we conjugated this protein to Quantum Dots (QDot^®^s). As stated previously, the QDot^®^s are carboxylated and in the presence of DCC, this fluorescent probe can form bonds with proteins or peptides, such as BMP-2. This powerful technique will provide critical fluorescent details regarding the binding activity of BMP-2 within the BMSCs. As noted, in 15- and 20-month-old mice, BMP-2 is not functioning properly in the BMSCs. However, whether BMP-2 can bind to BMPRs or is endocytosed into the cells is unclear and must be elucidated. Here, we isolated BMSCs from both the 6-month and 15-month-old B6 mice. These cells were treated with 100 µM MβCD for 24 h, an agent that depletes membranous cholesterol to prevent endocytosis, followed by stimulation with BMP-2-QDot^®^s for 6 h or left US. The cells were then immunostained for the nuclei, BMPRIa, and BMP-2-QDot^®^s, which will fluoresce green when present. As demonstrated in Figure 3 and Figure 4, all the BMSCs of 6-month-old mice express BMPRIa, whereas only the BMP-2-QDot^®^s-treated cells display green. Furthermore, the green is confined to BMPRIa, suggesting that the conjugation specifically targets and binds only to this receptor. Next, it is visually notable that within all the treatment groups, there is an increase in BMPRIa localization in the plasma membrane of the 15-month-old cells compared to the 6-month-old cells, except for in the MβCD only and BMP-2-QDot^®^s + MβCD-treated groups. Furthermore, the BMSCs of the 15-month-old mice did not display effective BMP-2-QDot^®^s binding; however, when these cells were treated with MβCD, the binding of BMP-2-QDot^®^s to BMPRIa significantly increased.

**Figure 5:**
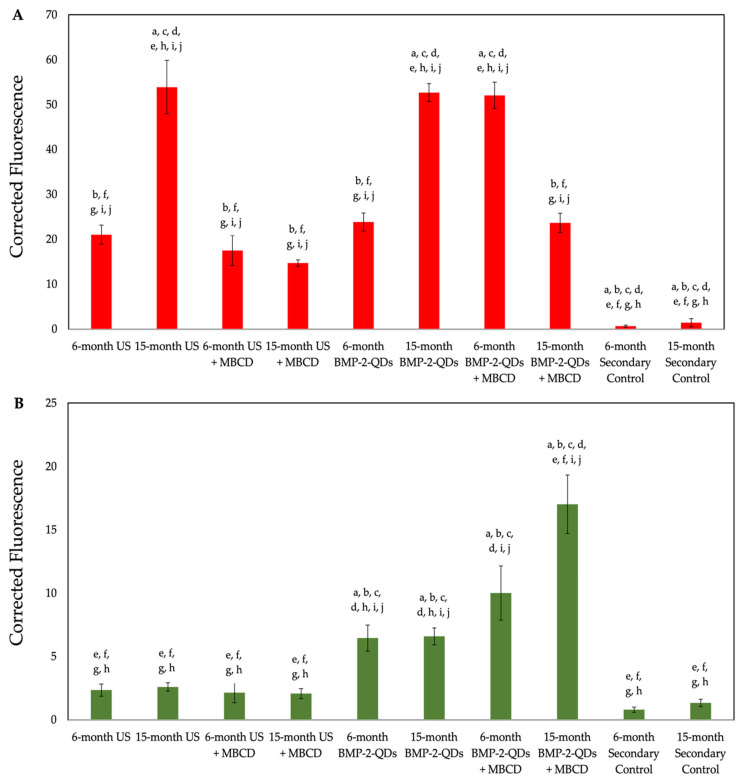
Quantification of immunostaining conducted on BMSCs. To further assess the increase or decrease in BMPRIa localization and BMP-2 binding, the images acquired from the confocal microscopy were semi-quantified. Fluorescence was calculated with at least 10 cells that were imaged and analyzed in ImageJ. The fluorescence from the cells was averaged for both BMPRIa and BMP-2-QDs. (**A**) The red fluorescence of BMPRIa was measured across all the conditions of both 6- and 15-month-old B6 mice. (**B**) The green fluorescence of BMPRIa was measured across all the conditions of both the 6- and 15-month-old B6 mice. The SEM bars are displayed above each bar. All the data were analyzed in ImageJ and statistical analyses were performed with the Tukey–Kramer HSD test. Statistical significance was set to *p* ≤ 0.05 and is displayed as lettering; here, the letters represent group one as the letter “a”, group two corresponds to the letter “b”, and so on. For example, if there is a letter “a” above a bar, that means this group is statistically different from group one.

Next, we semi-quantified these data by obtaining 10 random images and measuring the fluorescence of red and green within all the groups (Figure 5). As shown, the 15-month-old US cells and BMP-2-QDot^®^s only treated cells displayed an increase in BMPRIa localization at the cellular membrane when compared to the 6-month-old US BMSCs. Interestingly, treatment with MβCD and BMP-2-QDot^®^s in the 15-month-old cells slightly decreased the localization of BMPRIa at the membrane compared to the other conditions in this age group. In addition, as shown in Figure 4 treatment with MβCD increased the fluorescence of BMP-2-QDot^®^s when compared to the other 15-month conditions. Thus, these data suggest that BMPRIa is mis-localized and may be rescued by a cellular uptake inhibitor, such as MβCD.

### 3.3. MβCD-Treated Cells Displayed an Increase in Mineralization After BMP-2 Stimulation Compared to Untreated Cells

**Figure 6:**
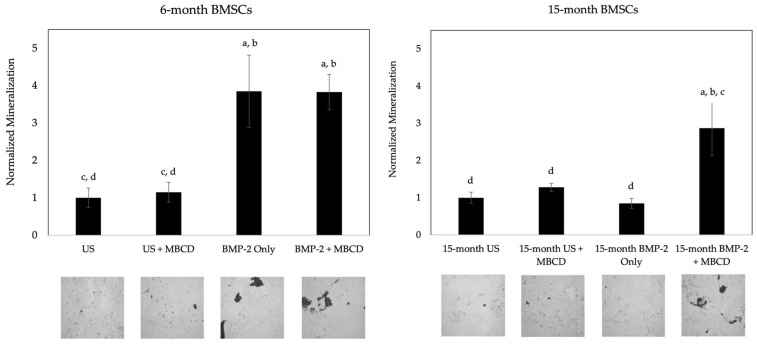
Von Kossa assay of the MβCD-treated BMSCs isolated from the 6- and 15-month-old mice. The BMSCs were obtained from the 6- and 15-month-old female B6 mice and treated with MβCD or left untreated. Afterward, the cells were stimulated with BMP-2 or left US. BMP-2 enhanced mineralization in the 6-month-old cells in both MβCD-treated and untreated cells, whereas in 15-month-old cells, only BMP-2 + MβCD led to mineralization. Random images were obtained from each condition and analyzed in ImageJ. Representative images are displayed underneath each bar. The error bars represent SEM and significance was determined using the Tukey–Kramer-HSD test. Statistical significance was set to *p* ≤ 0.05 and is displayed as lettering; here, the letters represent group one as the letter “a”, group two corresponds to the letter “b”, and so on. For example, if there is a letter “a” above a bar, that means this group is statistically different from group one.

As noted in the previous section, MβCD was able to disrupt the endocytosis of membrane domains, allowing for the possible rescue of BMPRIa mis-localization. Specifically, we unraveled that BMP-2 conjugated to QDot^®^s is successfully able to bind to BMPRIa in the BMSCs of both the 6- and 15-month-old mice, and this co-localization increased in 15-month mice when the cells were treated with MβCD. While this co-localization was observed, it remains unclear whether this increased binding will also lead to the activation of BMP signaling. For this next experimentation, the BMSCs isolated from the 6- and 15-month-old female B6 mice were obtained and seeded into 24-well plates. The cells were treated with MβCD or left untreated for 24 h, and then stimulated with BMP-2 or left US for 5 days. Afterward, the cells were fixed and subjected to the von Kossa assay as described in the methods section. As shown in Figure 6, the BMSCs of the 6-month-old mice displayed a significant increase in mineralization after the BMP-2 treatment in both the MβCD and no MβCD conditions compared to the US group. However, the 15-month-old mice only responded positively to BMP-2 when the cells were also treated with MβCD. Representative images are displayed under each bar. These data further affirm that BMPRIa may be mis-localized, and MβCD rescues these receptors to allow them to bind to BMP-2 and activate downstream signaling pathways.

## 4. Discussion

Natural aging and the effects of senescence are becoming increasingly prevalent as the world’s aging population is growing. This process poses many disorders and causes aberrancies in several signaling pathways. These disrupted signaling pathways can cause metabolic, neurocognitive, and bone or cartilage disorders, such as OP. OP is a debilitating bone disorder that affects one in four women and one in five men over the age of 50 [5]. This disease is characterized by a lower-than-normal BMD that likely arises from an imbalance between osteoblasts and osteoclasts. The activation of osteogenic signaling pathways in osteoblasts is mediated by BMP-2, which was approved by the FDA in 2002 to be used as a therapeutic [79]. However, several side-effects were reported after the usage of BMP-2, such as hematoma formation, radiculitis, and increased osteolysis, as BMP-2 also enhances osteoclastogenesis [6,62,80,81]. Furthermore, it was demonstrated that the primary osteoblasts isolated from OP patients and BMSCs obtained from aged (15- and 20-month-old) B6 mice are unresponsive to BMP-2 stimulation [5,6]. Interestingly, there was an increase in BMPRIa expression in both of these groups, even though BMP-2 binding is aberrant. This unresponsiveness and upregulation of BMPRIa could contribute partly to OP and may arise from the mis-localization or improper shuttling of BMPRs on the surface of cells. Thus, we explored whether these receptors could be shuttled from incorrect domains and into the proper regions of the cells.

To ensure B6 mice are a reliable model to investigate aberrant BMP signaling, the expression of BMPRIa must be further established. We first confirmed that the BMSCs of the US 15-month-old B6 mice produce more BMPRIa compared to US 6-month-old mice via Western blotting and confocal microscopy, confirming previous data that was observed in osteoblasts isolated from OP patients (Figure 1 and Figure 2) [6,75]. It is well documented that if BMP-2 does not bind to BMPRs, there is not an induction of osteogenesis [1,82,83]. As such, if BMP-2 signaling becomes aberrant as humans age, it is possible that osteoinduction is decreased. This deficiency in osteogenesis may prompt osteoblasts and their precursors to upregulate proteins involved in BMP signaling, such as BMPRIa. Furthermore, while the receptor may be upregulated, the receptors themselves are mis-localized and restrict pathway activation by BMP-2. As such, the binding dynamics of BMP-2 to BMPRIa and the activation of the SMAD signaling pathway were further explored here.

The precise molecular action of BMP-2 in vivo and in vitro has remained elusive over the past few decades. In this present study, we attempt to fill this knowledge gap by conjugating BMP-2 to a fluorescent probe that was described previously [73]. With this approach, we determined the binding affinity of BMP-2 to BMPRIa with QDot^®^s. BMP-2 was conjugated to QDot^®^s and added to BMSCs that were treated or untreated with MβCD. MβCD is a pharmacological agent capable of preventing endocytosis and disrupting membrane domains, which may allow BMPRIa and/or BMPRII to shuttle out and resume proper signaling if this pathway is indeed aberrant. As depicted in Figure 3 and Figure 4, BMP-2-QDot^®^s effectively bound to BMPRIa in both the MβCD-treated and untreated groups compared to the US and secondary control groups. However, as shown, while BMP-2-QDot^®^s co-localized with BMPRIa, this effect was drastically increased after the MβCD treatment in both age groups. Furthermore, it is possible that because BMPRIa is more prevalent in 15-month-old BMSCs, there is an increase in the substrates where BMP-2-QDot^®^s can bind. Therefore, the activation of SMAD signaling pathways must be elucidated to determine the activity of BMP-2. Taken together, while BMPRIa is upregulated in the 15-month-old mice, BMP-2 is unable to effectively bind and possibly fails to activate downstream signaling. In addition, the construction of a BMP-2-QDot^®^s probe is a powerful technique that informs the present study of BMP-2’s molecular mechanisms and can be utilized in other research fields.

After the BMP signaling pathway is activated, there is an upregulation of proteins involved in bone mineralization, including alkaline phosphatase (ALP) and osteocalcin. Therefore, if the BMSCs are treated with MβCD and BMP-2, these cells should be able to secrete a mineralized mineralization. Here, we measured the signaling output of BMP-2 with or without MβCD via the von Kossa assay for mineralization. The von Kossa assay visualizes mineralization deposition by first binding silver nitrate to phosphate or calcium, and then exposing the cells to UV light. The mineralization deposits are representative of osteogenesis, as ALP cleaves pyrophosphate molecules to yield phosphate [84]. BMP-2 was able to induce mineralization in both the BMP-2 alone and BMP-2 + MβCD-treated cells in the 6-month-old mice (Figure 6). However, mineralization only occurred in the 15-month-old BMSCs when the cells were exposed to both BMP-2 and MβCD (Figure 6). These data provide evidence that BMP-2 ineffectively binds to BMPRIa, as it fails to activate downstream BMP signaling demonstrated in this study and in previous studies. While BMP-2 may be binding to BMPRIa in the 15-month-old BMSCs, this binding is likely abnormal as indicated by a lack of a mineralized matrix produced by these cells. Furthermore, we provide evidence that this signaling can be restored after treating the cells with MβCD. This suggests that aberrant BMP signaling is implicated in OP and osteogenesis may be restored with the pharmacological inhibition of endocytosis.

Taken together, these data demonstrate that BMPRIa is mis-localized on the plasma membrane of the BMSCs isolated from the 15-month-old B6 mice and contributes to aberrant BMP signaling as a result of aging. Furthermore, the receptors may be shuttled out of the improper domains after treatment with the MβCD treatment. These data confirm aberrant BMP signaling in aged B6 mice that are reflective of OP patients and propose a potential therapeutic to restore BMP signaling. In addition, these data provide evidence that BMP-2 loosely associates with BMPRIa in 15-month-old mice, but the downstream activation of BMP signaling does not occur. Therefore, our findings fill the gap regarding aberrant BMP-2 signaling and its implication in aging or OP. While the present study does not investigate RNA expression or the genetic regulation of the factors involved in aberrant BMP signaling, these data may uncover more factors involved in this process. It would be critical to investigate the BMP-2-QDot^®^s in vivo to confirm the findings represented here. Altogether, these data uncover a mechanism by which BMP signaling can be restored in aged mice that can inform future clinical treatments.

## Data Availability

Any data or material that support the findings of this study can be made available by the corresponding author upon request.
